# Anti-Nuclear Antibodies in Patients with Polycystic Ovary Syndrome before and after Laparoscopic Electrocauterization

**Published:** 2013-06

**Authors:** Alamtaj Samsami Dehaghani, Nazanin Karimaghaei, Mohammad Ebrahim Parsanezhad, Mahyar Malekzadeh, Mohammad Mehrazmay, Nasrollah Erfani

**Affiliations:** 1 Department of Obstetrics and Gynecology, School of Medicine, Shiraz University of Medical Sciences, Shiraz, Iran;; 2 Shiraz Institute for Cancer Research, School of Medicine, Shiraz University of Medical Sciences, Shiraz, Iran

**Keywords:** Anti-nuclear autoantibodie, Polycystic ovary syndrome, Laparoscopic ovarian

## Abstract

Polycystic ovary syndrome (PCOS) has been suggested to be linked with autoimmune processes. Laparoscopic ovarian electrocauterization has the potency to stimulate more autoimmune reactions in PCOS patients. In the present study, we considered anti-nuclear antibodies (ANAs) as the hallmark of autoimmune reactions, and investigated the serum level of these antibodies in 35 patients with PCOS (21-38 years old) pre and one-month after electrocauterization, and in 35 fertile healthy women (25-35 years old) as the control group. Serum levels of ANAs, as well as ANA subtyping, were investigated using the Enzyme-Linked Immunosorbent Assay (ELISA). While 3 out of the 35 patients (8.6%) were positive for ANAs before electrocauterization, none of the controls was positive. The number of ANA-positive cases increased following electrocauterization (3 out of 35 [8.6%] before vs. 10 out of 35 [28.6%] after the procedure). The main ANA subtype in the positive samples was SS-A. The higher ANA level among the PCOS patients suggests association of the disease with autoimmune reactions. Laparoscopic ovarian electrocauterization seems to increase the number of positive-ANA patients.

## Introduction

Polycystic ovary syndrome (PCOS) is a disease of unknown etiology, which affects approximately 5-10% of women of reproductive age.^1^ Investigations have indicated an association between PCOS and specific autoimmune diseases and autoantibody production.^2^^-^^4^ Although ovarian drilling by different methods has been indicated to increase ovulation and pregnancy rate in patients with PCOS,^5^ this procedure has the potency to stimulate more autoimmune reactions via tissue destruction and cell injury.^6^

Production of antinuclear antibodies (ANAs) is the hallmark of almost all autoimmune reactions. Inflammation, immune hyper-stimulation, and any procedure that is associated with tissue destruction might stimulate ANA production.^7^ To shed light on the issue of ANA production in PCOS patients and to investigate the effect of laparoscopic electrocauterization on the production of these autoantibodies, in the present study we aimed to evaluate the serum level of ANAs in patients with PCOS and healthy fertile women, and to compare the ANA level before and after laparoscopic ovarian electrocauterization.

## Subjects and Methods

Thirty-five individuals diagnosed with infertility and PCOS (age range of 25-35 years) were recruited as the study group. PCOS was diagnosed according to the European Society for Human Reproduction and Embryology (ESHRE)/American Society for Reproductive Medicine (ASRM) PCOS consensus workshop and Rotterdam.^8^ All the patients had received medical therapy such as Metformin (1500 mg/day for 3 months), Clomiphene citrate (150 mg/day from the fifth to ninth day of each cycle for 5 cycles), and Dexamethasone (0.5 mg/day for 1 month), but without any response. 

The exclusion criteria were patients’ response to drug therapy and patients’ decision not to continue study. The control group comprised 35 fertile healthy women in the age range of 21 to 38 years, who experienced at least one pregnancy without any history of pregnancy loss or abdominal surgery and whose last child was delivered (by normal vaginal delivery) within one year before the study. The healthy control subjects had normal hormonal assay such as LH, follicular stimulating hormone (FSH), prolactin and thyroid stimulating hormone (TSH). 

The two-puncture technique was used for laparoscopic surgery. The study protocol was approved by the Ethics Committee at Shiraz University of Medical Sciences and informed consent was obtained before sample collection.

The Enzyme-Linked Immunosorbent Assay (ELISA) (AESKULISA, Germany) was employed to evaluate the serum level of ANAs in the control samples and in the patients before and one month after electrocauterization. Subtypes of ANAs were determined in the samples with high titers of ANAs (pre- or postoperative) by using a ELISA kit which was able to determine eight ANA subtypes: U1-RNA, Sn-ANP/Sm, Sm, SS-A, SS-B, Scl-70, CenpB, and Jo-1 (AESKULISA, Germany). The intra-assay and inter-assay coefficients of variation were smaller than 6% for all the assays performed. SPSS software package (SPSS 16.0, Chicago, IL, USA) was used for data assembly and analysis. 

## Results

All the patients with PCOS had infertility for more than a year, 30 (85.7%) had hirsutism, 17 (46.7%) suffered from acne, and 17 (46.7%) had obesity. Whereas 3 out of the 35 patients (8.6%) were positive for ANAs before electrocauterization, none of the controls was positive. The number of ANA-positive cases rose following electrocauterization (3 out of 35 [8.6%] before vs. 10 out of 35 [28.6%] after the procedure). 

Of the ANA-positive samples, ten samples (three samples before electrocauterization and 7 after electrocauterization) were studied for ANA subtyping. One case that became positive for ANAs after electrocauterization revealed to have all the 8 different subtypes of ANAs in her postoperative sample, while another one disclosed to have only SS-A subtype. In addition, two cases that were positive both in pre and postoperative settings, as well as a preoperative positive case that became negative postprocedurally, revealed to have SS-A subtype only. The other 5 samples were in the negative ranges for ANA subtypes. 

## Discussion

The results of the present study showed a high number of ANA-positive cases among patients with PCOS in comparison to healthy fertile subjects. Elevated serum levels of autoantibodies, including anti-histone, anti-dsDNA, and smooth muscle antibodies (SMAs), have already been reported in PCOS.^9^^,^^10^ Production of ANAs in PCOS might be the result of the activation of self-reaction responses to intracellular antigens, suggestive of an autoimmune etiology in the pathogenesis of PCOS. 

Chiming in with this finding, there are plenty of data available in the existing literature which link PCOS to autoimmune markers.^9^^,^^10^ Increased inflammatory responses and over expression of immune modulators have already been reported in these patients.^11^ Fulghesu et al.^12^ recently reported that monocytes of patients with insulin-resistant PCOS produce significant amounts of interleukin-6 (IL6), a potent inflammatory cytokine, in response to lipopolysaccharide (LPS). Low levels of progesterone in patients with PCOS have also been suggested to be another reason for immune over-stimulation.^13^ These data, in conjunction with our finding, suggest that patients with PCOS have raised levels of ANAs, most likely in consequence of immune hyperactivation and inflammation increment. Further studies seem to be necessary to determine the significance of ANAs in these patients.

In laparoscopic ovarian drilling, a laser or electrocautery is used to destroy parts of the ovaries. Our results demonstrated that following ovary electrocauterization, the number of ANA-positive cases had an increase. Although the exact mechanism remains to be elucidated, this finding may imply that the manipulation of the ovarian tissue and/or cauterization-induced inflammation may release the normally-occult nuclear antigens and consequently augment autoimmune reactions. It has already been established that cell injury may be followed by autoimmune reactions, although it does not necessarily end with autoimmune disease.^6^ Cell necrosis has also been well documented to be associated with the release of inflammatory mediators and immune stimulatory cytokines.^14^ Interestingly, the accumulation of platelets at the site of tissue injuries seems to be a powerful tool to stimulate immune activation.^15^ Alborzi et al.^16^ evaluated anti-ovarian antibodies (AOA) in Clomiphene-resistant PCOS patients before and after electrocauterization. Although their raw data indicated a trend toward rising AOA levels postprocedurally, normalization of the data based on the kit recommendation did not verify the significant production of AOA after laparoscopic ovarian electrocauterization.

In the present study, the most common ANA subtype among the positive individuals was SS-A, although of the 10 positive samples that underwent ANA subtyping, five were negative for all the subtypes in the subtyping experiment. This observation may come from the fact that the total ANA detected in the total ANA detection kit was divided for separate detection in the subtyping kit, resulting in a negative subtyping result from a sample with a positive total ANA. 

The findings of the present study collectively not only revealed a high ANA production in some patients with PCOS, but also suggested that laparoscopic ovarian electrocauterization might have exposed ovarian antigens to the immune system and consequently stimulated autoimmune reactions in the patients. The limitations of the study, including the low sample size and the qualitative nature of the ELISA kits, however, should not be ignored. 
